# Sudden Cardiac Death in Dialysis: Arrhythmic Mechanisms and the Value of Non-invasive Electrophysiology

**DOI:** 10.3389/fphys.2019.00144

**Published:** 2019-02-25

**Authors:** Dimitrios Poulikakos, Katerina Hnatkova, Sofia Skampardoni, Darren Green, Philip Kalra, Marek Malik

**Affiliations:** ^1^Renal Department, Salford Royal NHS Foundation Trust, Salford, United Kingdom; ^2^Centre for Cardiac Research, Institute of Cardiovascular Sciences, The University of Manchester, Manchester, United Kingdom; ^3^National Heart and Lung Institute, Imperial College London, London, United Kingdom

**Keywords:** sudden cardiac death, arrhythmias, dialysis, QRS-T angle, TCRT, heart rate variability, implantable loop recorders

## Abstract

Sudden Cardiac Death (SCD) is the leading cause of cardiovascular death in dialysis patients. This review discusses potential underlying arrhythmic mechanisms of SCD in the dialysis population. It examines recent evidence from studies using implantable loop recorders and from electrophysiological studies in experimental animal models of chronic kidney disease. The review summarizes advances in the field of non-invasive electrophysiology for risk prediction in dialysis patients focusing on the predictive value of the QRS-T angle and of the assessments of autonomic imbalance by means of heart rate variability analysis. Future research directions in non-invasive electrophysiology are identified to advance the understanding of the arrhythmic mechanisms. A suggestion is made of incorporation of non-invasive electrophysiology procedures into clinical practice.

**Key Concepts**:

– Large prospective studies in dialysis patients with continuous ECG monitoring are required to clarify the underlying arrhythmic mechanisms of SCD in dialysis patients.

– Obstructive sleep apnoea may be associated with brady-arrhythmias in dialysis patients. Studies are needed to elucidate the burden and impact of sleeping disorders on arrhythmic complications in dialysis patients.

– The QRS-T angle has the potential to be used as a descriptor of uremic cardiomyopathy.

– The QRS-T angle can be calculated from routine collected surface ECGs. Multicenter collaboration is required to establish best methodological approach and normal values.

– Heart Rate Variability provides indirect assessment of cardiac modulation that may be relevant for cardiac risk prediction in dialysis patients. Short-term recordings with autonomic provocations are likely to overcome the limitations of out of hospital 24-h recordings and should be prospectively assessed.

## Introduction

Chronic kidney disease affects 5–7% of the global population and is associated with a 10-fold increase in cardiovascular mortality. Over 2 million people worldwide are on renal replacement therapy (Couser et al., 2011). Despite technological advances in the field of renal replacement therapy, the long-term survival of patients receiving chronic dialysis remains poor, comparable with survival in some forms of cancer (United States Renal Data System, 2012). Cardiovascular disease and infections account for most of these deaths. SCD accounts for a large proportion of cardiovascular deaths and almost one fourth of the overall mortality in these patients as evidenced from registry data (United States Renal Data System, 2012) and from prospective trials with death adjudication (Cheung et al., 2004; Wanner et al., 2005). High risk of SCD is present even at early stages of CKD but it increases substantially when patients are on HD. This high risk is aggravated by the fluctuations in volume and electrolyte status induced by the intermittent pattern of the treatment (Poulikakos et al., 2014a).

It has been shown that renal dysfunction increases the risk of ventricular arrhythmias in patients with underlying ischaemic heart disease. After a first myocardial infarction, the presence of CKD stage 3 was found to be associated with a sixfold increase in odds of developing VF independently of traditional cardiovascular risk factors (Dalal et al., 2012). Advanced CKD has been shown to be a strong predictor of appropriate delivery of shock therapies for ventricular arrhythmias in implantable cardioverter defibrillator recipients (Robin et al., 2006; Cuculich et al., 2007). In these patients, advanced CKD has also been associated with decreased time to the first appropriate shock (Hreybe et al., 2006). However, the implantation of cardioverter defibrillators based on existing guidance has not been associated with survival benefit in retrospective studies in this high-risk population (Charytan et al., 2011; Pun et al., 2015).

Although CKD often coexists with coronary artery disease (Herzog et al., 2011), SCD is also highly prevalent in dialysis patients without a history of coronary artery disease or impaired left ventricular ejection fraction (Baigent et al., 2011). Possible mechanisms that increase cardiovascular risk related to advanced CKD include the ensuing bone mineral abnormalities and vascular calcification (London et al., 2003), endothelial dysfunction (Poulikakos et al., 2014d), electrolyte fluxes (Wang et al., 2018), chronic inflammation (Nowak and Chonchol, 2018), insulin resistance (Semple et al., 2011), increased levels of FGF 23 (Gutiérrez et al., 2009; Isakova et al., 2011) and autonomic imbalance (Converse et al., 1992b). Based on epidemiological data, it has also been postulated that heritable factors contribute to the risk of cardiac arrest in patients receiving dialysis (Chan et al., 2015). However, the exact pathogenic mechanisms that interlink these factors and lead to the high risk cardiovascular phenotype in CKD remain elusive.

Furthermore, whereas the increased risk of ventricular arrhythmias due to advanced CKD in the context of severe ischaemic cardiomyopathy has been confirmed (Robin et al., 2006; Cuculich et al., 2007; Dalal et al., 2012; Wan et al., 2014), the underlying arrhythmic mechanisms leading to SCD in dialysis patients with preserved left ventricular function are not well understood. Recent studies that used ILR in small cohorts of asymptomatic HD patients have shown increased rates of bradyarrhythmic events and deaths (Kalra et al., 2018).

Risk stratification strategies to identify dialysis individuals who are at high risk of SCD are lacking. This is despite them attending hospital or satellite facilities for their dialysis treatment three times weekly and having clinical observations and blood tests more often than any other out-patient group. Because of this, it is recognized that non-invasive electrophysiology could serve the unmet clinical need for risk prediction in this population (Skampardoni et al., 2018b). Descriptors of repolarization aberration and autonomic dysregulation can be assessed by applying advanced computerized analysis of snapshot and/or continuous ECGs and can be used for this purpose. There is accumulating evidence that increased QRS-T angle is a good descriptor of the arrhythmogenic myocardial substrate and predicts cardiovascular risk in this population (Skampardoni et al., 2018b). Furthermore, measurements of cardiac autonomic regulation by HRV have also shown promising results in cardiac risk assessment in dialysis patients.

This review summarizes the recent evidence from the use of ILRs in HD patients and electrophysiological studies in animal models of CKD, exploring the potential underlying arrhythmic mechanisms. It also presents the evidence from the use of non-invasive electrocardiography in dialysis patients focusing on QRS-T angle and HRV and concludes with suggestions for future directions of research.

### Arrhythmic Patterns in Dialysis Patients. Insight From Studies Involving Continuous ECG Monitoring From Implantable Loop Recorders

Since 2015, five studies using ILR in asymptomatic HD patients have been published (Silva et al., 2015; Wong M.C. et al., 2015; Roberts et al., 2017; Roy-Chaudhury et al., 2018; Sacher et al., 2018) (Table 1). Collectively, they included 317 prevalent HD patients with largely preserved left ventricular ejection fraction. The age ranged from 56 ± 12 (Roy-Chaudhury et al., 2018) to 68 ± 12 (Roberts et al., 2017) years and follow up time ranged from 6 (Roy-Chaudhury et al., 2018) to 21 ± 7 (Sacher et al., 2018) months. In total, there were 52 deaths (15%) of which 21 (6.6% of patients, 40% of deaths) were classified as SCD. ILRs revealed bradyarrhythmia or asystole as the terminal rhythm in the majority (13) of SCD deaths. SCD cases in the 5 studies ranged from 83% (Wong M.C.G. et al., 2015) to 25% (Roberts et al., 2017) of all deaths and bradycardia as the terminal SCD rhythm was reported in 42% (Silva et al., 2015) to 100% (Sacher et al., 2018) of SCD cases. On the contrary, there were only 4 cases of sustained VT or VF across the 5 studies. In total 9 patients required pacemaker implantation for bradyarrhythmia across the 5 studies [3/30 patients in the study by Roberts et al. (2017), 5/66 in the study by Roy-Chaudhury et al. (2018), and 1/50 in the study by Wong M.C. et al. (2015)]. The temporal pattern of arrhythmias in the detailed study by Roy-Chaudhury et al. (2018) showed an increased rate of clinically significant bradycardias, defined as rate <40 bpm for ≥6 s, toward the latter stages of the long interdialytic interval. One of the studies (Roberts et al., 2017) reported on the circadian pattern of the bradycardic episodes noting an increased rates during night-time. The proportion of patients with at least one episode of severe bradycardia ranged from 20% (Roy-Chaudhury et al., 2018) to 25% (Silva et al., 2015) and asystolic events ranged from 4% (Silva et al., 2015) to 20% (Wong M.C.G. et al., 2015). The studies reported high rates of AF ranging from 13% (Silva et al., 2015) to 40.9% (Roy-Chaudhury et al., 2018).

**Table 1:** Studies using implantable loop recorders in HD patients.

Study	Number	Age (years)	IHD	Follow up (months)	SCD/Total mortality (%)	Brady-arrhythmic SCD	VT-VF	Significant arrhythmic events	Comments
Wong M.C.G. et al., 2015	50	67 ± 11	48%	18 ± 4	8/10 (80%)	6	2	**Pacemaker in 1 patient** Bradycardia 30% Sinus arrest 28% 2nd degree atrioventricular block 4% Non-sustained VT 20% AF 28%	All SCDs and increased rates of arrhythmias during long interdialytic interval.
Silva et al., 2015	100	59 ± 8.8	34%	14 ± 4	7/18 (39%)	3	1	Bradycardia 25% Asystole 4% Non-sustained VT 56% AF 13%	Left Ventricular dilatation associated with higher occurrence of non-sustained VT.
Sacher et al., 2018	71	65 ± 9	NR	21 ± 7	4/16 (25%)	4	–	Conduction Abnormalities 14% (patient years) Non-sustained VT 9% (patient years) AF 37% (20% *de novo*)	
Roberts et al., 2017	30	68 ± 12	22%	18 ± 12	2/8 (25%)	0	1	**Pacemakers in 3 patients** (2 dual chamber pacemakers and 1 biventricular pacemaker)	Increased nocturnal bradycardias.
Roy-Chaudhury et al., 2018	66	56 ± 12	48.5%	6	0	–	–	**Pacemakers in 5 patients** Bradycardia 25.8% Asystolic events^∗^ 10.6% Non-sustained Ventricular arrhythmias ^∗^ 77.3% AF^∗^ 40.9% (device detected)	Increased rates of bradyarrhythmic events at the end of long interdialytic interval.

*AF, atrial fibrillation; IHD, ischemic heart disease; NR, not reported; SCD, sudden cardiac death; VT, ventricular tachycardia; VF, ventricular fibrillation*. *Table was modified with permission by Kalra et al. (2018).*

Although these studies provide novel information, this should be interpreted with caution. The scarcity of available information about the prevalence of bradyarrhythmias from long-term monitoring in asymptomatic patients need to be taken into consideration.

Previous studies using 24-h recordings in healthy individuals have shown a high prevalence of bradycardias (heart rate 40 beats/min or less) during sleep at 24% in males aged 22–27 years (Brodsky et al., 1977). With advancing age, this decreased to 3% in subjects aged 40–79 years (Bjerregaard, 1983) and 2% in the group aged 60–85 years (Fleg and Kennedy, 1982). ILRs are likely to detect more bradycardic events than 24 h Holter monitors (Simantirakis et al., 2004) but have not been used to study healthy populations.

The participants in the HD ILR studies were not young and, despite the lack of knowledge regarding the normal incidence of bradycardias in HD patients and notwithstanding the small number of relatively low cardiovascular risk participants and the heterogeneity amongst the studies, these investigations show a high burden of bradyarrhythmias in HD patients with preserved ejection fraction. This potentially contributes substantially to the overall burden of SCD. Consistent with the HD ILR studies, a recent epidemiological study that investigated registry data of 28,471 dialysis patients (Wang et al., 2016) showed that HD patients had an almost sixfold increased incidence of requiring pacemaker insertion compared to matched patients with normal renal function.

### Pathogenesis of Bradycardia

Elucidating the underlying mechanisms of bradycardias and their relationship to SCD has important clinical implications relating to the required routine screening, to the decisions of potential use of beta blockers, and/or to considerations of potential interventions such as pacemaker insertion.

Electrolyte shifts, especially pre-dialysis hyperkalaemia, have been implicated as potential triggers of bradyarrhythmias particularly at the end of the long interdialytic interval in a typical 3 dialysis session week. The electrophysiological role of hyperkalaemia in triggering bradyarrhythmias is likely potentiated by the presence of underlying structural abnormalities in the conduction system and/or abnormal cardiac autonomic regulation of the pacemaker activity that prevent adequate heart rate reactions in response to physiological stress. Cardiac valve calcification may affect the cardiac conduction system and has also been implicated in bradyarrhythmias (Mainigi et al., 2012). It is highly prevalent in HD (Matsuo et al., 2018) and peritoneal dialysis (PD) patients (Wang et al., 2003) and has been associated with increased cardiovascular mortality (Wang et al., 2018). In addition, evidence of impaired cardiac autonomic modulation in dialysis patients has been demonstrated in several HRV studies (Poulikakos et al., 2014a).

The potential contribution of obstructive sleep apnoea to bradyarrhythmias in dialysis patients merits attention. Sleep apnoea is typically characterized by episodes of bradycardia (Zwillich et al., 1982) as shown with long term recordings with ILR in this population (Simantirakis et al., 2004). Sleep apnoea is highly prevalent in HD patients occurring in 34.5% (Tada et al., 2007) to 65% (Sakaguchi et al., 2011) of cases. It is commonly underdiagnosed and its severity has been associated with the magnitude of fluid overload (Ogna et al., 2015). Interestingly, bradyarrhythmias due to sleep apnoea do not seem to be potentiated by the administration of beta blockers (Wolf et al., 2016) and can be successfully treated with continuous positive airway pressure treatment (Simantirakis et al., 2004). No information regarding the presence of sleep apnoea was provided in the publications of the HD ILR studies. However, it can be speculated that it was present in a fair proportion of the participants and that sleep apnoea related bradyarrhythmias would tend to increase during periods of increased fluid retention, i.e., at the end of the long interdialytic interval.

### Atrial Fibrillation and Sudden Cardiac Death

The high rate of AF in the HD population is another important finding and may be linked to increased risk of SCD. Evidence suggests that AF may be associated with SCD in cardiac patients (Gronefeld et al., 2000; Berton et al., 2009; Pedersen et al., 2006) and in the general population (Chen et al., 2013). Several mechanisms have been suggested linking AF with SCD (Chen et al., 2014). It has been speculated that fast ventricular rate in the context of atrial tachyarrhythmia can reduce ventricular refractoriness (Denes et al., 1974) thus increasing the vulnerability to ventricular arrhythmias. In addition, AF can lead to short-long-short sequences in the ventricular cycle length that in turn predispose to some types of ventricular arrhythmias (Gronefeld et al., 2000).

## Arrhythmic Mechanisms in Animal Models of CKD

Animal models have been used to investigate the pre-arrhythmic substrate and the mechanisms of arrhythmias in CKD (Table 2).

**Table 2:** Electrophysiological studies in animal models of CKD.

Study	Animal model	Electrophysiological study	Time	Fibrosis assessment	Time	Main findings
Hsueh et al., 2014	Cy/+ rats (12) compared to normal rats (9)	Optical mapping Induced VF via pacing	35 weeks	Picrosirius red staining	35 weeks	Longer action potential duration at 80% repolarization and cycle length thresholds to induce alterans in CKD rats. VF induced in 9/12 CKD rats vs. 2/9 normal rats. Electrical remodeling favoring initiation and maintenance of VTs in CKD rats. No difference in cardiac fibrosis.
Lee et al., 2017	Unilateral nephrectomy at 8 weeks rats (6) compared to sham operated rats (6)	Surface ECG Patch-clamp studies	8 weeks after nephrectomy	Trichrome staining and picrosirius red staining^∗^ Cellular hypertrophy based on cellular capacitance in ventricular cardiomyocytes	8 weeks post nephrectomy	Prolonged QTc in CKD rats. Epicardial prolongation of action potential duration in CKD rats and decreased transmural gradient in CKD rats compared to controls. Electronegative LDL may underlie downregulation of KChIP2 protein expression. No difference in fibrosis.
Zhao et al., 2016	Cy/+ rats (6) Compared to normal rats (8)	Continuous ECG Subcutaneous nerve activity recordings	Started at 35 weeks and continued for 23 ± 14 days	Trichrome staining H&E staining	40 weeks for normal rats After sudden death at CKD rats (35 weeks+ 23 ± 14 days)	6 deaths due to bradycardia or atrioventricular block. 3 deaths due to VT/VF. Myocardial calcification involving the conduction system. Blunted response of heart rate to subcutaneous nerve activity increase in CKD rats. Sudden death preceded by reduction in subcutaneous nerve activity.
Fontes et al., 2015	Aged mice deoxycorticosterone acetate and high salt diet (*n* = 7) & 5/6 -subtotal nephrectomy and high salt diet (5) Controls (4)	Programmed electrical stimulation of arrhythmias Conduction velocity from electrocardiograms	68 weeks 11 weeks	Picrosirius red staining	68 weeks 11 weeks	Arrhythmias induced in 86 and 75% in hearts from aged mice and partially nephrectomised mice on high salt diet compared to 0% in control hearts. Cx43 expression was reduced and interstitial fibrosis was increased in both groups with renal dysfunction.

*CKD, chronic kidney disease; Cx43, connexin 43, gap junction protein expressed in the heart; ECG, electrocardiogram; VT, ventricular tachycardia; VF, ventricular fibrillation. ^∗^Experiments on cardiac fibrosis were performed in previous study of the same group using the same CKD rat model.*

Hsueh et al. (2014) used a rat cystic kidney disease model (Cy/+ rats) characterized by progressive CKD that reaches terminal uraemia in about 40 weeks. They investigated 12 CKD and 9 normal rats at week 35 with detailed electrophysiological studies including optical mapping and a pacing protocol for VF induction. CKD rats showed increased action potential duration and longer cycle length thresholds to induce action potential duration alterans compared to normal rats, indicating increased vulnerability to ventricular arrhythmias. VF was induced in 9/12 (75%) CKD rats compared to 2/9 (22%) normal rats. Examination of the heart tissue following euthanasia revealed upregulation of pro-fibrotic pathways in CKD animals but did not show significant difference in cardiac fibrosis between the two groups.

Lee et al. (2017) used a rat model of mild CKD (unilateral nephrectomy at 8 weeks) and performed electrophysiological studies 8 weeks after the operation, including surface ECG. After euthanasia, cardiomyocytes were isolated for further electrophysiological recordings of ionic currents and membrane potential. CKD rats exhibited prolonged QTc compared to normal rats. Longer action potential duration at 50 and 90% repolarization of paced cardiomyocytes was observed in epicardiac cardiomyocytes in CKD rats compared to normal rats but there was no difference in endocardial cardiomyocytes. The transient outward potassium current *I*_to_ was reduced in epicardial cardiomyocytes in CKD rats compared to controls, and associated with decreased transmural gradient of *I*_to_. Similar to the previous study, increased heterogeneity of repolarization occurred independently of the cardiac fibrosis that was observed at similar levels in CKD and normal rats (Chang et al., 2015).

(Zhao et al. (2016) used the same CKD model as presented by Hsueh et al. (2014) (Cy/+ rats). The animals were implanted with ECG and subcutaneous nerve activity electrode recorders at 35 weeks. Subcutaneous nerve activity has been shown to correlate with the stellate ganglion nerve activity and was thus used to estimate the sympathetic tone (Robinson et al., 2015). All 6 CKD rats died suddenly 23 ± 14 days after the implantation. All SCDs occurred after the development of atrioventricular block. Terminal rhythms were progressive bradycardia and asystole in 3 rats, VF in 2 rats and VT converted to bradycardia in one rat. In contrast to the previous studies, histology showed fibrosis in all CKD rats, especially surrounding the AV node and in the sub-endocardium, and calcification affecting the conduction system. These findings were not present in normal rats. The magnitude of the increase in heart rate corresponding to increase in subcutaneous nerve activity was smaller in CKD rats 5 days prior to death.

Fontes et al. (2015) used two different mouse models of renal impairment. First model included aged mice that received deoxycorticosterone acetate for 8 weeks and a high-salt diet that continued for 35 weeks. Second model dealt with adult mice that underwent 5/6-subtotal nephrectomy and were treated for 11 weeks with a high-salt diet. *Ex vivo* epicardial mapping was used to assess vulnerability to arrhythmias and hearts were assessed for fibrosis and characterized for connexin 43 (Cx43). Both models demonstrated a high incidence of arrhythmias accompanied by increased interstitial fibrosis and decreased Cx43 expression in the heart.

The first two animal investigations (Hsueh et al., 2014; Lee et al., 2017) suggest that electrophysiological aberrations leading to increased repolarization heterogeneity and vulnerability to ventricular arrhythmias occur before the development of overt cardiac fibrosis and calcification which is in turn associated with conduction system disorders and bradyarrhythmias in advanced CKD in rodents. Considering the two studies (Hsueh et al., 2014; Zhao et al., 2016) that used the same Cy/+ CKD models, Hsueh et al. (2014) did not find fibrosis in heart tissue retrieved following euthanasia at 35 weeks whereas Zhao et al. (2016) detected fibrosis and calcification in heart tissue retrieved 3 weeks later (after death), i.e., in animals exposed to CKD for longer.

The observation that the relationship between subcutaneous nerve activity, a surrogate of sympathetic tone, and heart rate was different in CKD rats compared to normal rats is in keeping with human studies describing abnormal autonomic cardiac modulation in CKD.

However, fundamental electrophysiological differences exist between rodents and large mammals (Sallam et al., 2015), making rodents a poor model of human electrophysiology. Furthermore, these animal models did not study the dynamic interactions of the fluctuations in fluid and electrolyte changes. Therefore their contribution in understanding the mechanisms of SCD in dialysis patients is limited. Thus, interpretation and extrapolations from these animal experiments need to be made with caution.

## Non-Invasive Electrophysiology in Dialysis Patients

The standard 12 lead ECG is an essential clinical tool in CKD and dialysis patients (Skampardoni et al., 2018b). It is commonly used for the assessment of the acutely unwell patient. It can also be used as routine investigation for the detection of conduction abnormalities that require clinical intervention such as with medication or dialysis prescription review, further studies and referral for pacemaker implantation. However, the fluctuant fluid and electrolyte status of HD patients may affect the ECG waveform that in turn can influence the automated ECG interpretations including interval calculations (Poulikakos and Malik, 2016). This influence poses challenges in using automated ECG measurements in risk stratification studies. Furthermore, important repolarization abnormalities usually referred to as “non-specific T wave changes” are only subjectively defined and cannot be studied in a quantifiable manner. Advanced computerized analysis of the ECG has the potential to overcome these limitations.

### Repolarization Heterogeneity and the Case for QRS-T Angle Assessment in CKD

**FIGURE 1:**
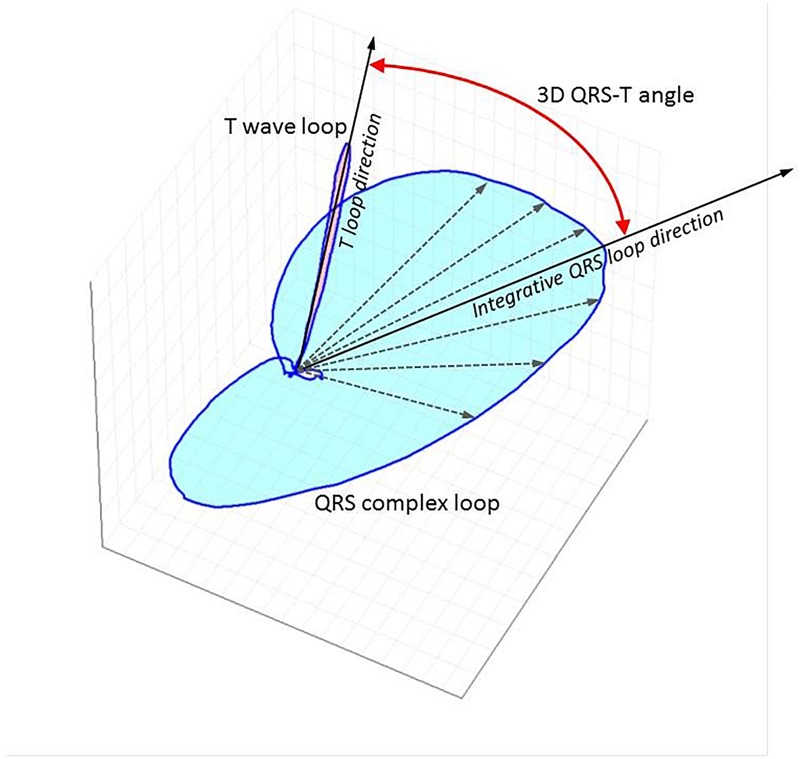
Three-dimensional representation of QRS and T vector loops following computerized analysis of standard 12 lead ECG using singular value decomposition to yield an orthogonal lead system (TCRT method). The little white circle in the middle depicts the loop of the p wave. The curved red line with arrow at both ends depicts the spatial QRS-T angle. The maximal T wave vector magnitude is used for the calculations with this method. TCRT is the averaged cosine of the angles between the T wave vector and all vectors within the QRS complex exceeding a pre-defined level. A different method of QRS-T angle measurement from standard 12 lead digital snapshot ECG uses inverse Dower matrix for vectrographic transformation and calculates the angle between the mean vectors of the QRS and T wave. QRS-T angle has also been calculated from continuous signal averaged ECGs as the angle between the peak QRS vector and the peak T vector.

The QRS-T angle is an established marker of global repolarization heterogeneity (Waks et al., 2016; Hnatkova et al., 2017) that can be measured from a standard 12 lead ECG (Figure 1). It is based on three-dimensional vectorial representation of the electrical activity of the heart, that is not affected by impedance changes due to fluid shifts (Vitolo et al., 1987). The QRS-T angle is calculated numerically, is reproducible (Poulikakos et al., 2013; Hnatkova et al., 2017) and thus suitable for risk stratification purposes. A larger QRS-T angle has been shown to predict SCD (de Bie et al., 2013b; Tereshchenko et al., 2016), cardiovascular mortality (Skampardoni et al., 2018a) and all cause mortality (Tereshchenko et al., 2016; de Bie et al., 2013b; Poulikakos et al., 2018) in different cohorts of predominantly African (Tereshchenko et al., 2016) and Caucasian (Sallam et al., 2015; Poulikakos et al., 2018; Skampardoni et al., 2018a) dialysis patients. However, one study in 325 Taiwanese HD patients (Lin et al., 2007) did not find a significant association between magnitude of QRS-T angle and outcomes. Instead, this study reported that T wave residuum, a descriptor of regional repolarization heterogeneity (Zabel and Malik, 2002), was an independent predictor of cardiovascular mortality and SCD. Studies that have investigated the prognostic value of the QRS-T angle in dialysis patients are summarized in Table 3.

**Table 3:** Studies of QRS-T angle for risk prediction in dialysis patients.

Study	Participants	Age (years)	IHD	Calculation	Follow up	Outcome
de Bie et al., 2013b	277 (prevalent) Male 62.1% HD 62.8% Peritoneal dialysis 37.2%	56.3 ± 17.0	24.5%	Inverse Dower matrix from standard 12 lead snapshot ECG. **Measured as angle between mean QRS and T vectors.**	60 months	QRS-T angle independent predictor of all-cause mortality (*N* = 91) and SCD (*N* = 18).
Tereshchenko et al., 2016	358 (incident HD) Male 59% Black 73%	55 ± 13	37%	Calculated from continuous unfiltered averaged *xyz* ECG signal. **Measured as angle between spatial mean QRS vector and spatial peak T vector.**	864.6 person-years of follow-up	QRS-T angle associated with all-cause mortality (*N* = 77) cardiovascular mortality (*N* = 35) and SCD (*N* = 15).
Skampardoni et al., 2018a	178 (prevalent HD) Male 72% Caucasian 80% South Asian 17% Black 3%	67 ± 14	23.5%	Singular value decomposition to standard snapshot 12 lead ECG. **Measured as total cosine R to T.**	36 ± 19 months	TCRT independent predictor of cardiac deaths (*N* = 17) and major cardiac events (*N* = 54).
Poulikakos et al., 2018	72 (prevalent HD) Males 58% Caucasian 44% Black 31% South Asians 25%	61 ± 15	21%	Singular value decomposition to overlapping 10 s segment 12 lead ECGs during continuous monitoring repeated 5 times every 2 weeks. **Measured as total cosine R to T.**	54.8 months	TRCT independent predictor of all-cause mortality (*N* = 16) and major cardiac events (*N* = 9).
Lin et al., 2007	325 (incident) HD Male 44% Taiwanese population	64 ± 13	19%	Singular value decomposition to standard snapshot 12 lead ECG. **Measured as total cosine R to T**	25 ± 22 months	TRCT not associated with all-cause mortality (*N* = 154) cardiovascular mortality (*N* = 79) or arrhythmia related deaths (*N* = 59). T wave residuum independent predictor for all outcomes.

*ECG, electrocardiogram; F/up, follow up; HD, hemodialysis; IHD, Ischemic heart disease; SCD, sudden cardiac death; TCRT, Total Cosine R to T.*

**FIGURE 2:**
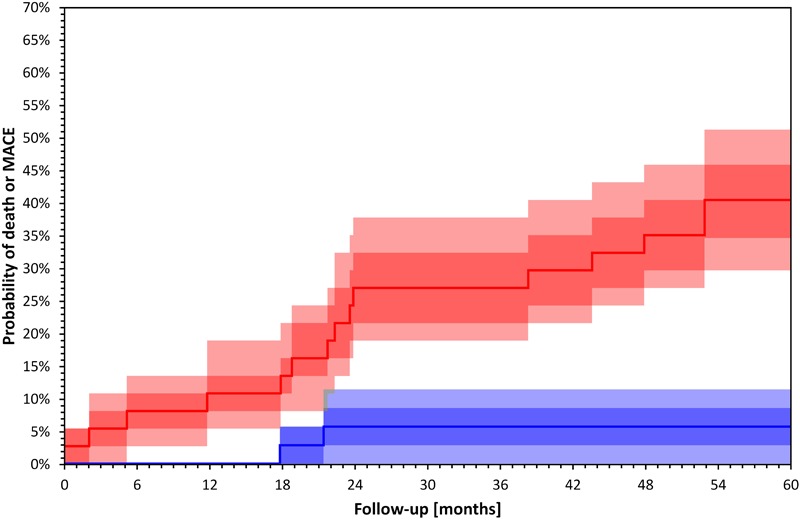
Kaplan–Meier survival curves of total mortality and major cardiac events 72 HD patients included in a recently published study (Poulikakos et al., 2018) stratified by QRS-T angle calculated by TCRT above (red) and below (blue) median value (*p* = 0.000 Log Rank test). Darker bands depict interquartile ranges and the lighter bands ranges between 10th and 90th percentiles. The calculation of confidence intervals was performed using bootstrap 10,000 repetitions. Major cardiac events were defined as sudden cardiac death, acute coronary syndrome, coronary revascularization or admission due to heart failure or arrhythmia.

An increased QRS-T angle has been associated with echocardiographic left ventricular hypertrophy (Tereshchenko et al., 2016), decreased global longitudinal strain (Skampardoni et al., 2018a), lower left ventricular ejection fraction, and with left ventricular systolic dyssynchrony (de Bie et al., 2013a) in different cohorts of HD patients. It has also been associated with increased coronary artery calcium burden, and elevated Troponin T levels in patients receiving peritoneal dialysis (Jaroszynski et al., 2009). The impact of HD treatment on the QRS-T angle has been variable (Jaroszynski et al., 2010; Poulikakos et al., 2013). The intra-subject stability of the QRS-T angle at the start and the end of dialysis has been demonstrated in a cohort of 72 HD patients who underwent five intra-dialytic Holter ECGs at 2-week intervals (Poulikakos et al., 2013). Studies reporting on associations of the QRS-T angle with echocardiographic characteristics and intradialytic changes are presented in Table 4. Figure 2 shows an example of Kaplan–Meier event probability curves of major cardiac events and overall mortality in 72 HD patients (Poulikakos et al., 2018) stratified by QRS-T angle above and below median value.

**Table 4:** Studies of QRS-T angle reporting on associations with cardiovascular parameters and dialysis procedure.

Study	Participants	Age (years)	IHD	Calculation of QRS-T angle	Findings
de Bie et al., 2013a	101 prevalent Male 76% 66% HD, 44% Peritoneal Dialysis	56.3 ± 17	30%	Inverse Dower matrix from standard 12 lead snapshot ECG. **Measured as angle between mean QRS and T vectors.**	QRS-T angle associated with left ventricular ejection fraction, QRS duration and left ventricular systolic dyssynchrony.
Tereshchenko et al., 2016	358 incident HD Male 59% Black 73%	55 ± 13	37%	Calculated from continuous unfiltered averaged *xyz* ECG signal. **Measured as angle between spatial mean QRS vector and spatial peak T vector.**	QRS-T angle >75° associated with wider QRS and echocardiographic left ventricular hypertrophy defined as left ventricular mass index >51 g/m^2.7^ in men and >47 g/m^2.7^ in women.
Skampardoni et al., 2018a	178 prevalent HD Male 72% Caucasian 80% South Asian 17% Black 3%	67 ± 14	23.5%	Singular value decomposition to standard snapshot 12 lead ECG. **Measured as total cosine R to T.**	QRS-T angle by TCRT correlated with left ventricular mass indexed for height in univariate and Global Longitudinal Strain in multivariate analysis.
Jaroszynski et al., 2009	57 prevalent Peritoneal Dialysis patients 49% male Caucasians	47.7 ± 7.1	Excluded	Inverse Dower matrix from standard 12 lead snapshot ECG. **Measured as angular difference between maximum spatial QRS and T vectors.**	QRS-T angle associated with increased coronary artery calcium burden, atherosclerosis and troponin T elevation.
Jaroszynski et al., 2010	73 prevalent HD 52% male Caucasians	51.5 ± 4.5	Not reported	Inverse Dower matrix from standard 12 lead snapshot ECG. **Measured as angular difference between maximum spatial QRS and T vectors.**	QRS-T angle associated with Troponin T. HD session resulted in increased QRS-T in 59 patients, decreased in 12 patients, and unchanged in 2 patients.
Poulikakos et al., 2013	72 (prevalent HD) Males 70%	61 ± 15	21%	Singular value decomposition to overlapping 10 s segment 12 lead ECGs during continuous monitoring repeated 5 times every 2 weeks. **Measured as total cosine R to T.**	Intra-subject reproducibility confirmed using analysis of variance. Variable effect of HD to TCRT Intradialytic QRS-T change correlated with PTH levels.

*ECG, electrocardiogram; HD, hemodialysis; IHD, Ischemic heart disease; TCRT, total Cosine R to T*.

### Methodological Considerations

Different groups have used different methods for the calculation of the QRS-T angle that are summarized in Table 3. The methodological differences stem from different approaches in characterizing the spatial deviation between the vectorial loops of depolarization (QRS) and repolarization (T wave). These differences may partially explain the different numerical cut-off values (Hnatkova et al., 2017) used in predicting outcomes in dialysis populations. The challenge lies mainly in the fact that the loops can be irregular and curved in space. It has recently been demonstrated in cardiac patients that the discrepancies between the various methods increase as the angle becomes more abnormal and the distortion of the vectors increases (Hnatkova et al., 2017). In a recent comparative study in cardiac patients, the calculation of the QRS-T angle by the so-called TCRT method was more reliable for cardiac risk prediction. It is thought that the calculation of the sum of the cosines between QRS and T wave vectors increases the ability of depicting the shape and orientation irregularities that accompany increased repolarization heterogeneity.

Racial, and previously described sex differences (Smetana et al., 2002) should also be taken into consideration in studies measuring the QRS-T angle. For example, in the Taiwanese HD cohort there was no association between QRS-T angle and cardiac outcomes whereas there was with T wave residuum; this may reflect racial differences in repolarization heterogeneity patterns and QRS-T angle. In a previous population study in healthy subjects, the QRS-T angle in Taiwanese individuals was significantly different compared to African, Caucasian and Indian healthy subjects (Elaine and Macfarlane, 2016).

The mechanisms linking abnormal repolarization heterogeneity with clinical outcomes and cardiac structure and function abnormalities remain unclear and will need to be assessed in prospective studies.

### Autonomic Dysfunction and Heart Rate Variability in CKD

Sympathetic overactivation has short and long-term proarrhythmogenic effects and may play a role in the pathogenesis of arrhythmic deaths in CKD patients. Sympathetic activation impacts on cellular channel activity increasing repolarization heterogeneity (Vaseghi et al., 2012) and plays a role in the pathogenesis of cardiac fibrosis via α-adrenergic receptor stimulation and via triggering inflammatory responses (Levick et al., 2010). Autonomic imbalance decreases myocardial ability to suppress disorganized electrical activation and may compromise the heart pacemaker response to challenges.

Sympathetic nerve activity, assessed by microneurography of the peroneal nerve, increases with declining renal function (Grassi et al., 2011) and is elevated in patients with end-stage renal disease (Converse et al., 1992b). Potential contributing underlying mechanisms of sympathetic overactivation in CKD (Kaur et al., 2017) include elevated angiotensin 2 levels (Siddiqi et al., 2009) that potentiate norepinephrine release from sympathetic nerve terminals, stimulation of afferent renal nerves and chemoreceptors by adenosine release due to renal ischaemia (Biaggioni et al., 1991; Vallon et al., 2006), and reduced nitric oxide (NO) availability (Jourde-Chiche et al., 2011) potentiated by upregulation of MicroRNA-92a in dialysis patients (Young et al., 2009; Shang et al., 2017).

The baroreflex regulation has also been shown to be impaired in CKD. In dialysis patients, impaired baroreflex sensitivity was shown to be associated with vascular calcification and arterial stiffness (Chesterton et al., 2005) indicating a potential anatomical link between vascular changes and the function of baroreceptors.

Direct measurement of the autonomic system with microneurography poses practical challenges and is not suitable for clinical practice. Nevertheless, indirect measurements are possible by assessing HRV (Task Force of the European Society of Cardiology and North American Society of Pacing and Electrophysiology, 1996). HRV is a non-invasive method based on analysis of tachograms of continuous ECGs. It assesses the cardiac autonomic regulation based on the differences in cardiac pacemaker responses to autonomic activations. HRV can provide an approximation of the cardiac autonomic regulation that integrates multiple feedback systems. In addition to traditional time and frequency domain measurement several different methodologies have been developed (Task Force of the European Society of Cardiology and North American Society of Pacing and Electrophysiology, 1996; Sassi et al., 2015).

Many groups have used HRV methodology in dialysis patients. A pubmed search using the terms HRV and dialysis returns 122 studies with the first one published already in 1986 (Forsstrom et al., 1986). Abnormal HRV indices have been associated with all cause (Hayano et al., 1999; Oikawa et al., 2009; Suzuki et al., 2012; Chen et al., 2016; Ng et al., 2017; Poulikakos et al., 2018) and cardiovascular mortality (Hayano et al., 1999; Fukuta et al., 2003; Oikawa et al., 2009; Chen et al., 2016) in east Asian HD patients and in one small study including predominantly Caucasian HD patients (Skampardoni et al., 2018a). Depressed HRV has also been associated with all cause (Pei et al., 2015; Chiang et al., 2016) and cardiovascular (Hayano et al., 1999) mortality in east Asian patients receiving peritoneal dialysis.

Frequent daily HD has been shown to improve components of HRV (Chan et al., 2014). Preserved residual urine output has also been associated with different HRV profiles in peritoneal dialysis patients (Tang et al., 2012) and depressed HRV has been associated with increased PTH (Zhang et al., 2013, 2015; Poulikakos et al., 2014b; Jiang et al., 2016) and FGF 23 levels (Zhang et al., 2015). Depressed HRV has also been associated with increased pulse pressure (Poulikakos et al., 2014c) in patients with end stage renal disease and with increased pulse wave velocity in patients with CKD stages 3–5 (Chandra et al., 2014).

Despite the consistent results confirming the value of HRV in CKD patients, major challenges exist in extrapolating associations from these studies and incorporating HRV measurements into clinical practice. The main challenge is related to the sensitivity of HRV to environmental influences and the need for strict standardization, particularly for 24-h ECG recordings, which is practically impossible when the recording is performed in the outpatient setting.

### Short Term HRV Measurements During Autonomic Provocations in HD Patients

For the purposes of standardized assessment, several studies used short term recordings around or during the dialysis procedure, i.e., a period with relatively standardized environment characterized by volume and electrolyte shifts that provoke the autonomic system in a predictable manner. It is known that patients who exhibit excessive intradialytic decline in systolic blood pressure have higher risk of cardiovascular and overall mortality (Chou et al., 2018; Reeves and Mc Causland, 2018). The pathogenesis of intradialytic hypotension is multifactorial (Reeves and Mc Causland, 2018) but it has been postulated that abnormal activation of the sympathetic system plays a central role (Converse et al., 1992a). Some studies have shown that spectral HRV assessment during intradialytic recordings can differentiate patients who are prone to intradialytic hypotension (Severi et al., 1997; Hernando et al., 2015). However, Barnas et al. (1999) reported similar intradialytic HRV profiles between hypotension prone and stable patients up to the moment of the hypotensive episode where they observed bradycardia and decline in LF power.

**FIGURE 3:**
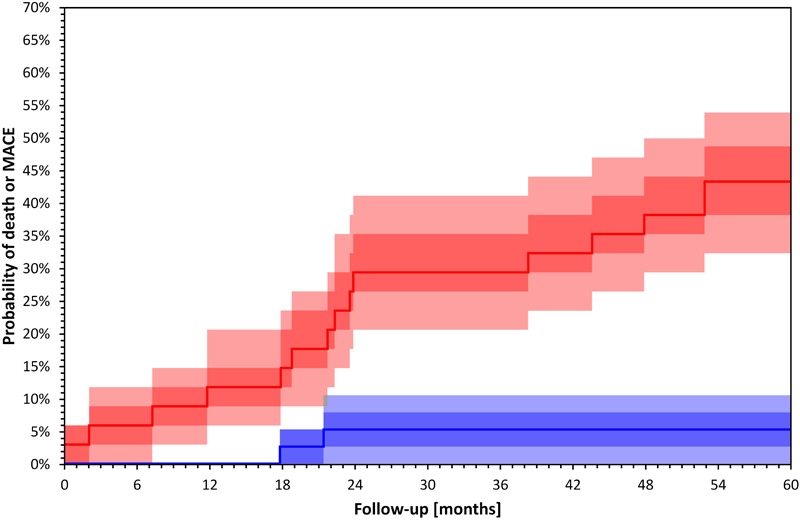
Kaplan–Meier survival curves of total mortality and major cardiac events in 72 HD patients included in a recently published study (Poulikakos et al., 2018) stratified by LF/HF above (blue) and below (red) median value (*p* = 0.001 Log Rank test). Darker bands depict interquartile ranges and the lighter bands ranges between 10th and 90th percentiles. The calculation of confidence intervals was performed using bootstrap 10,000 repetitions. Major cardiac events were defined as sudden cardiac death, acute coronary syndrome, coronary revascularization or admission due to heart failure or arrhythmia.

Studies using short term intradialytic ECG monitoring have shown that HRV parameters are influenced by the rate of volume removal during dialysis (Tsuji et al., 2017) and by the dialysis modality between HD and hemofiltration (Genovesi et al., 2007). Two studies also reported associations between short term HRV measurement during dialysis and mortality (Chen et al., 2016; Poulikakos et al., 2018). In a recent study using intradialytic HRV assessment depressed LF/HF over the first hour of treatment predicted overall mortality (Poulikakos et al., 2018). Chen et al. (2016) used spectral HRV assessment before and after HD and showed that decreased difference in LF was associated with increased risk of overall and cardiovascular mortality. Figure 3 shows an example of Kaplan–Meier event probability curves for major cardiac events and overall mortality in a cohort of 72 HD patients (Poulikakos et al., 2018) stratified by LF/HF ratio above and below median value.

Few studies have used short term HRV assessment during autonomic postural, provocations in HD patients. In the study by Genovesi et al. (2007) postural provocation including 10 min standing before dialysis was performed. Interestingly, whilst active standing was associated with increase in normalized LF and decrease in normalized HF during HD treatment, this was not the case when treatment with hemofiltration was used. Echeverria et al. (2017) showed an increase in LF/HF in response to postural provocations before and after HD treatment.

Genetic differences might contribute to the differences in reported HRV response to autonomic provocations. A recent study in 114 HD patients (Ribas Ribeiro et al., 2018) suggested that genetic polymorphism in the angiotensin converting enzyme, an enzyme that modulates the autonomic system, determines the autonomic response during HD. In this study, an increase in LF and LF/HF through dialysis was observed only in patients with II angiotensin converting enzyme genotype and not in patients with DD genotype. Studies using short term HRV assessment with autonomic provocations are presented in Table 5.

**Table 5:** Studies using short term Heart Rate Variability assessment with autonomic provocations.

Study	Number of HD patients	Setting	Outcome
Severi et al., 1997	14 stable and 14 hypotension- prone	Spectral HRV from continuous intradialytic ECG.	Spectral HRV different in hypotension prone vs. stable patients.
Hernando et al., 2015	24 patients 52 HD treatment sessions	Spectral HRV from continuous intradialytic ECG and continuous intradialytic arterial BP signal.	Spectral HRV parameters during the first 30 min of the treatment session different in hypotension prone vs. stable patients.
Barnas et al., 1999	11 stable and 9 hypotension prone patients	Spectral HRV from continuous intradialytic ECG and continuous intradialytic arterial BP signal.	No difference in groups up to the point of hypotensive episodes when LF HRV declined
Tsuji et al., 2017	35 patients divided in 3 groups based on UFR	Spectral HRV and approximate entropy from continuous intradialytic ECG.	LF/HF values and approximate entropy increased at the end of HD in patients with UFR >15 ml/h/kg.
Genovesi et al., 2007	10 patients Treated with 6 session of HD and 6 sessions of Hemofiltration	Spectral HRV from continuous ECG during pre-HD postural provocation (10 min rest and 10 min active standing) followed by intradialytic measurements (10 min periods every hour and after the end of treatment) and continuous arterial BP signal.	Normalized LF was higher and HF lower in HF compared to HD. Active standing was associated with increase in normalized LF and decrease in normalized HF in HD but not in Hemofiltration.
Chen et al., 2016	72 patients 5 sessions every 2 weeks	Spectral HRV from continuous intradialytic ECG.	Low LF/HF during the first hour associated with overall mortality. Intrasubject stability of HRV parameters averaged over the first and last hour confirmed by repeated measure ANOVA.
Chen et al., 2016	182 patients	Spectral HRV from 5?min ECG before and 30 min after HD.	Decreased difference in LF associated with increased overall and cardiovascular mortality.
Echeverria et al., 2017	19 stable patients 20 healthy controls	Continuous ECG recording during 16 min in supine position, and during 16 min of active orthostatism pre and post HD treatment for HD patients. Last 5 min of the recording at each position were used for HRV.	Increase in LF/HF and in the short-term scaling exponent (a1) on standing before and after HD.
Ribas Ribeiro et al., 2018	114	HRV from continuous ECG measured for 20 min before, in the second hour and immediately after HD. Assessment of ACE genotypes.	Homozygosity for the II allele of the ACE gene associated with greater increase in LF and LF/HF during HD compared to DD genotype.

*ACE, angiotensin converting enzyme; BP, blood pressure; D, deletion; ECG, electrocardiogram; HD, hemodialysis; HF, high frequency; LF, low frequency power; HRV, heart rate variability; I, insertion; UFR, ultrafiltration rate.*

Two small studies have reported reasonable reproducibility of HRV measurements from intradialytic recordings (Poulikakos et al., 2014c; da Silva et al., 2016).

## Conclusion-Future Directions

### Bradyarrhythmic and Tachyarrhythmic Risk

Recent ILR studies have shown an important relative contribution of bradyarrhythmias to sudden death in dialysis patients. However, these studies did not include incident dialysis patients with high cardiovascular risk that may be more vulnerable to ventricular tachyarrhythmias. Future large studies with long term ECG monitoring in incident dialysis patients are needed to elucidate the precise incidence and underlying arrhythmic mechanisms of SCD. These studies should aim at developing risk stratification strategies for both tachyarrhythmic and bradyarrhythmic risks. Tachyarrhythmic risk is mainly related to static and dynamic ventricular repolarization abnormalities and it is possible that repolarization patterns and changes related dialysis induced fluid and electrolyte shifts prior to the fatal arrhythmias may be important for its prediction. On the contrary, bradyarrhythmic risk is mainly related to cardiac periodicity and is likely to be influenced by autonomic system abnormalities including sleep apnoea.

The ECG signals should be regularly interrogated during the course of such studies and treatment for significant bradyarrhythmias should be offered. Non-invasive surface long term ECG technology with sufficient memory that can record continuous high quality ECG signal could serve the dual purpose of potentially fatal or final rhythm detection and data acquisition for risk profiling for both tachyarrhythmias and bradyarrhythmias. In addition, non-invasive continuous long term ECG monitoring devices that can be attached and exchanged during a dialysis visit may facilitate the recruitment of high risk incident patients who may not wish to attend additional hospital visits or undergo invasive procedures.

Research protocols should include detailed cardiovascular, genetic and sleep disorder phenotyping.

These studies, in addition to clarifying the prevalence of bradyarrhythmias and ventricular arrhythmias, will provide important information about the burden of AF and its potential association with SCD. An association between AF and increased risk of SCD has been shown in cardiac patients (Chen et al., 2014) and may be relevant in the dialysis population given the extremely high burden of AF.

### QRS-T Angle-Need for Standardization and Integration Into Clinical Practice

The QRS-T angle assessment has substantial potential to be used as a descriptor of uremic cardiomyopathy. It can also be easily incorporated into clinical practice. In the first instance, the different methodological approaches need to be rigorously tested and compared in existing large ECG databases of CKD and dialysis patients to determine the best methodological approach to the angle expression. This should be based on a multicenter collaboration between clinical research teams and bioengineers. Such collaborative effort can help determine normal reference values and increase our understanding of racial and gender differences in the CKD and dialysis populations.

Furthermore, it is important for the clinical and research community and industry partners in ECG manufacturing to understand the importance of collecting high quality ECGs and storing them in easily accessible formats (Malik et al., 2018) in order to create and maintain open access ECG databases. Such databases would facilitate meaningful research that in turn can inform clinical practice about the possibilities of reducing cardiovascular mortality. The possibility and practical requirements of complementing the existing national and/or international (Goodkin et al., 2001; Perl et al., 2014) renal databases with repositories of routinely collected digital ECG should be explored.

### HRV-Methodological Challenges and Clinical Practicality

In the field of HRV and assessment of cardiac autonomic modulation, further research is required to standardize the measurements in dialysis patients. Short-duration tests with autonomic provocations could overcome methodological shortcomings of 24-h outpatient ambulatory ECG recordings. These assessments can be organized around the dialysis treatment in order to minimize a patient’s inconvenience and be suitable to be incorporated into regular clinical practice in the future. HRV measurements may be useful for both bradyarrhythmic and tachyarrhythmic risk stratification. The value of cardiac autonomic modulation assessment during standardized autonomic provocations for prediction of significant bradyarrhythmias should be prospectively investigated.

On the other hand, ECG acquisition during autonomic provocations has the potential to yield important prognostic information for tachyarrhythmias, while at the same time characterizing cardiac autonomic regulation measured by HRV and by dynamic repolarization profiles. Short duration continuous ECG measurements with standardized controlled provocations can be used to assess dynamic changes of the QRS-T angle during different stages of cardiac autonomic status; this has been shown to strengthen risk prediction in cardiac patients (Kentta et al., 2012). This type of measurement has the potential to shed more light on the links between autonomic status and cardiac electrical activity and explore the value of combined electrophysiological assessment in this group of patients.

Non-invasive electrophysiology has an important role in CKD patients. Close collaboration across the disciplines of biomedical engineering, clinical cardiology and nephrology is required to bridge the gaps between available technological tools, research efforts and clinical translation in arrhythmic complications of CKD.

## Author Contributions

DP: concept, design, drafting article, and critical revision of article. KH and SS: critical revision of article. DG and PK: concept and critical revision of article. MM: concept, design, and critical revision of article.

## Conflict of Interest Statement

The authors declare that the research was conducted in the absence of any commercial or financial relationships that could be construed as a potential conflict of interest.

## Abbreviations

AF: atrial fibrillation
CKD: chronic kidney disease
ECG: electrocardiogram
HD: hemodialysis
HRV: heart rate variability
IHD: ischemic heart disease
ILR: implantable loop recorder
SCD: sudden cardiac death
TCRT: total Cosine R To T
VF: ventricular fibrillation
VT: ventricular tachycardia

## References

[B1] Baigent C. Landray M. J. Reith C. Emberson J. Wheeler D. C. Tomson C. (2011). The effects of lowering LDL cholesterol with simvastatin plus ezetimibe in patients with chronic kidney disease (Study of Heart and Renal Protection): a randomised placebo-controlled trial. *Lancet* 377 2181–2192. 10.1016/S0140-6736(11)60739-3 21663949 PMC3145073

[B2] Barnas M. G. Boer W. H. Koomans H. A. (1999). Hemodynamic patterns and spectral analysis of heart rate variability during dialysis hypotension. *J. Am. Soc. Nephrol.* 10 2577–2584. 10589697 10.1681/ASN.V10122577

[B3] Berton G. Cordiano R. Cucchini F. Cavuto F. Pellegrinet M. Palatini P. (2009). Atrial fibrillation during acute myocardial infarction: association with all-cause mortality and sudden death after 7-year of follow-up. *Int. J. Clin. Pract.* 63 712–721. 10.1111/j.1742-1241.2009.02023.x 19392921

[B4] Biaggioni I. Killian T. J. Mosqueda-Garcia R. Robertson R. M. Robertson D. (1991). Adenosine increases sympathetic nerve traffic in humans. *Circulation* 83 1668–1675. 10.1161/01.CIR.83.5.1668 2022024

[B5] Bjerregaard P. (1983). Mean 24 hour heart rate, minimal heart rate and pauses in healthy subjects 40-79 years of age. *Eur. Heart J.* 4 44–51. 10.1093/oxfordjournals.eurheartj.a061370 6339245

[B6] Brodsky M. Wu D. Denes P. Kanakis C. Rosen K. M. (1977). Arrhythmias documented by 24 hour continuous electrocardiographic monitoring in 50 male medical students without apparent heart disease. *Am. J. Cardiol.* 39 390–395. 10.1016/S0002-9149(77)80094-5 65912

[B7] Chan C. T. Chertow G. M. Daugirdas J. T. Greene T. H. Kotanko P. Larive B. (2014). Effects of daily hemodialysis on heart rate variability: results from the Frequent Hemodialysis Network (FHN) Daily Trial. *Nephrol. Dial. Transplant.* 29 168–178. 10.1093/ndt/gft212 24078335 PMC3888308

[B8] Chan K. E. Newton-Cheh C. Gusella J. F. Maddux F. W. (2015). Heritability of risk for sudden cardiac arrest in ESRD. *J. Am. Soc. Nephrol.* 26 2815–2820. 10.1681/ASN.2014090881 25882830 PMC4625678

[B9] Chandra P. Sands R. L. Gillespie B. W. Levin N. W. Kotanko P. Kiser M. (2014). Relationship between heart rate variability and pulse wave velocity and their association with patient outcomes in chronic kidney disease. *Clin. Nephrol.* 81 9–19. 10.5414/CN108020 24356038 PMC4504149

[B10] Chang K. C. Lee A. S. Chen W. Y. Lin Y. N. Hsu J. F. Chan H. C. (2015). Increased LDL electronegativity in chronic kidney disease disrupts calcium homeostasis resulting in cardiac dysfunction. *J. Mol. Cell. Cardiol.* 84 36–44. 10.1016/j.yjmcc.2015.03.016 25871829

[B11] Charytan D. M. Patrick A. R. Liu J. Setoguchi S. Herzog C. A. Brookhart M. A. (2011). Trends in the use and outcomes of implantable cardioverter-defibrillators in patients undergoing dialysis in the United States. *Am. J. Kidney Dis.* 58 409–417. 10.1053/j.ajkd.2011.03.026 21664735

[B12] Chen L. Y. Benditt D. G. Alonso A. (2014). Atrial fibrillation and its association with sudden cardiac death. *Circ. J.* 78 2588–2593. 10.1253/circj.CJ-14-0814 25262841

[B13] Chen L. Y. Sotoodehnia N. Buzkova P. Lopez F. L. Yee L. M. Heckbert S. R. (2013). Atrial fibrillation and the risk of sudden cardiac death: the atherosclerosis risk in communities study and cardiovascular health study. *JAMA Intern. Med.* 173 29–35. 10.1001/2013.jamainternmed.744 23404043 PMC3578214

[B14] Chen S. C. Huang J. C. Tsai Y. C. Hsiu-Chin Mai R. N. Jui-Hsin Chen R. N. Kuo P. L. (2016). Heart rate variability change before and after hemodialysis is associated with overall and cardiovascular mortality in hemodialysis. *Sci. Rep.* 6:20597. 10.1038/srep20597 26854202 PMC4745005

[B15] Chesterton L. J. Sigrist M. K. Bennett T. Taal M. W. McIntyre C. W. (2005). Reduced baroreflex sensitivity is associated with increased vascular calcification and arterial stiffness. *Nephrol. Dial. Transplant.* 20 1140–1147. 10.1093/ndt/gfh808 15827047

[B16] Cheung A. K. Sarnak M. J. Yan G. Berkoben M. Heyka R. Kaufman A. (2004). Cardiac diseases in maintenance hemodialysis patients: results of the HEMO Study. *Kidney Int.* 65 2380–2389. 10.1111/j.1523-1755.2004.00657.x 15149351

[B17] Chiang J. Y. Huang J. W. Lin L. Y. Chang C. H. Chu F. Y. Lin Y. H. (2016). Detrended fluctuation analysis of heart rate dynamics is an important prognostic factor in patients with end-stage renal disease receiving peritoneal dialysis. *PLoS One* 11:e0147282. 10.1371/journal.pone.0147282 26828209 PMC4734614

[B18] Chou J. A. Streja E. Nguyen D. V. Rhee C. M. Obi Y. Inrig J. K. (2018). Intradialytic hypotension, blood pressure changes and mortality risk in incident hemodialysis patients. *Nephrol. Dial. Transplant.* 33 149–159. 10.1093/ndt/gfx037 28444336 PMC5837776

[B19] Converse R. L. Jr. Jacobsen T. N. Jost C. M. Toto R. D. Grayburn P. A. Obregon T. M. (1992a). Paradoxical withdrawal of reflex vasoconstriction as a cause of hemodialysis-induced hypotension. *J. Clin. Invest.* 90 1657–1665. 1430196 10.1172/JCI116037 PMC443221

[B20] Converse R. L. Jr. Jacobsen T. N. Toto R. D. Jost C. M. Cosentino F. Fouad-Tarazi F. (1992b). Sympathetic overactivity in patients with chronic renal failure. *N. Engl. J. Med.* 327 1912–1918. 1454086 10.1056/NEJM199212313272704

[B21] Couser W. G. Remuzzi G. Mendis S. Tonelli M. (2011). The contribution of chronic kidney disease to the global burden of major noncommunicable diseases. *Kidney Int.* 80 1258–1270. 10.1038/ki.2011.368 21993585

[B22] Cuculich P. S. Sánchez J. M. Kerzner R. Greenberg S. L. Sengupta J. Chen J. (2007). Poor prognosis for patients with chronic kidney disease despite ICD therapy for the primary prevention of sudden death. *Pacing Clin. Electrophysiol.* 30 207–213. 10.1111/j.1540-8159.2007.00651.x 17338717

[B23] da Silva D. M. Macedo M. C. Lemos L. B. Vieira F. C. Pirôpo U. S. Andrade H. B. (2016). Reliability analysis of the heart autonomic control parameters during hemodialysis sessions. *Biomed. Tech.* 61 623–630. 10.1515/bmt-2015-0239 27010774

[B24] Dalal D. de Jong J. S. Tjong F. V. Wang Y. Bruinsma N. Dekker L. R. C. (2012). Mild-to-moderate kidney dysfunction and the risk of sudden cardiac death in the setting of acute myocardial infarction. *Heart Rhythm* 9 540–545. 10.1016/j.hrthm.2011.11.014 22079886

[B25] de Bie M. K. Ajmone Marsan N. Gaasbeek A. Bax J. J. Delgado V. Rabelink T. J. (2013a). Echocardiographical determinants of an abnormal spatial QRS-T angle in chronic dialysis patients. *Nephrol. Dial. Transplant.* 28 3045–3052. 10.1093/ndt/gft347 24092849

[B26] de Bie M. K. Koopman M. G. Gaasbeek A. Dekker F. W. Maan A. C. Swenne C. A. (2013b). Incremental prognostic value of an abnormal baseline spatial QRS-T angle in chronic dialysis patients. *Europace* 15 290–296. 10.1093/europace/eus306 23024335

[B27] Denes P. Wu D. Dhingra R. Pietras R. J. Rosen K. M. (1974). The effects of cycle length on cardiac refractory periods in man. *Circulation* 49 32–41. 10.1161/01.CIR.49.1.32 4271710

[B28] Echeverria J. C. Infante O. Perez-Grovas H. Gonzalez H. Jose M. V. Lerma C. (2017). Effects of orthostatism and hemodialysis on mean heart period and fractal heart rate properties of chronic renal failure patients. *Artif. Organs* 41 1026–1034. 10.1111/aor.12887 28548688

[B29] Elaine C. Macfarlane P. W. (2016). “Comparison of spatial QRS-T angle in different healthy racial groups,” in *Paper Presented at the 2016 Computing in Cardiology Conference* Vancouver.

[B30] Fleg J. L. Kennedy H. L. (1982). Cardiac arrhythmias in a healthy elderly population: detection by 24-hour ambulatory electrocardiography. *Chest* 81 302–307. 10.1378/chest.81.3.302 7056104

[B31] Fontes M. S. Papazova D. A. van Koppen A. de Jong S. Korte S. M. Bongartz L. G. (2015). Arrhythmogenic remodeling in murine models of deoxycorticosterone acetate-salt-induced and 5/6-subtotal nephrectomy-salt-induced cardiorenal disease. *Cardiorenal Med.* 5 208–218. 10.1159/000430475 26195973 PMC4478316

[B32] Forsstrom J. Heinonen E. Valimaki I. Antila K. (1986). Effects of haemodialysis on heart rate variability in chronic renal failure. *Scand. J. Clin. Lab. Invest.* 46 665–670. 10.3109/00365518609083729 3787165

[B33] Fukuta H. Hayano J. Ishihara S. Sakata S. Mukai S. Ohte N. (2003). Prognostic value of heart rate variability in patients with end-stage renal disease on chronic haemodialysis. *Nephrol. Dial. Transplant.* 18 318–325. 10.1093/ndt/18.2.318 12543887

[B34] Genovesi S. Bracchi O. Fabbrini P. Luisetto E. Viganò M. R. Lucini D. (2007). Differences in heart rate variability during haemodialysis and haemofiltration. *Nephrol. Dial. Transplant.* 22 2256–2262. 10.1093/ndt/gfm125 17400561

[B35] Goodkin D. A. Mapes D. L. Held P. J. (2001). The dialysis outcomes and practice patterns study (DOPPS): how can we improve the care of hemodialysis patients? *Semin. Dial.* 14 157–159. 10.1046/j.1525-139X.2001.00043.x 11422917

[B36] Grassi G. Quarti-Trevano F. Seravalle G. Arenare F. Volpe M. Furiani S. (2011). Early sympathetic activation in the initial clinical stages of chronic renal failure. *Hypertension* 57 846–851. 10.1161/HYPERTENSIONAHA.110.164780 21300663

[B37] Gronefeld G. C. Mauss O. Li Y. G. Klingenheben T. Hohnloser S. H. (2000). Association between atrial fibrillation and appropriate implantable cardioverter defibrillator therapy: results from a prospective study. *J. Cardiovasc. Electrophysiol.* 11 1208–1214. 10.1046/j.1540-8167.2000.01208.x 11083241

[B38] Gutiérrez O. M. Januzzi J. L. Isakova T. Laliberte K. Smith K. Collerone G. (2009). Fibroblast growth factor 23 and left ventricular hypertrophy in chronic kidney disease. *Circulation* 119 2545–2552. 10.1161/CIRCULATIONAHA.108.844506 19414634 PMC2740903

[B39] Hayano J. Takahashi H. Toriyama T. Mukai S. Okada A. Sakata S. (1999). Prognostic value of heart rate variability during long-term follow-up in chronic haemodialysis patients with end-stage renal disease. *Nephrol. Dial. Transplant.* 14 1480–1488. 10.1093/ndt/14.6.1480 10383012

[B40] Hernando D. Sornmo L. Sandberg F. Laguna P. Llamedo M. Bailon R. (2015). Identification of patients prone to hypotension during hemodialysis based on the analysis of cardiovascular signals. *Med. Eng. Phys.* 37 1156–1161. 10.1016/j.medengphy.2015.10.003 26525780

[B41] Herzog C. A. Asinger R. W. Berger A. K. David M. C. Javier D. Robert G. H. (2011). Cardiovascular disease in chronic kidney disease. A clinical update from Kidney Disease: improving Global Outcomes (KDIGO). *Kidney Int.* 80 572–586. 10.1038/ki.2011.223 21750584

[B42] Hnatkova K. Seegers J. Barthel P. Novotny T. Smetana P. Zabel M. (2017). Clinical value of different QRS-T angle expressions. *Europace* 20 1352–1361. 10.1093/europace/eux246 29016907 PMC6075511

[B43] Hreybe H. Ezzeddine R. Bedi M. Barrington W. Bazaz R. Ganz L. I. (2006). Renal insufficiency predicts the time to first appropriate defibrillator shock. *Am. Heart J.* 151 852–856. 10.1016/j.ahj.2005.06.042 16569548

[B44] Hsueh C. H. Chen N. X. Lin S. F. Chen P. S. Gattone V. H. Allen M. R. (2014). Pathogenesis of arrhythmias in a model of CKD. *J. Am. Soc. Nephrol.* 25 2812–2821. 10.1681/ASN.2013121343 24854269 PMC4243359

[B45] Isakova T. Xie H. Yang W. Xie D. Anderson A. H. Scialla J. (2011). Fibroblast growth factor 23 and risks of mortality and end-stage renal disease in patients with chronic kidney disease. *JAMA* 305 2432–2439. 10.1001/jama.2011.826 21673295 PMC3124770

[B46] Jaroszynski A. Czekajska-Chechab E. Drelich-Zbroja A. Zapolski T. Ksiazek A. (2009). Spatial QRS-T angle in peritoneal dialysis patients: association with carotid artery atherosclerosis, coronary artery calcification and troponin T. *Nephrol. Dial. Transplant.* 24 1003–1008. 10.1093/ndt/gfn581 18978067

[B47] Jaroszynski A. Wysokiński A. Bednarek-Skublewska A. Głowniak A. Książek P. Sodolski T. (2010). The effect of a single dialysis session on spatial QRS-T angle in haemodialysis patients. *Nephrol. Dial. Transplant.* 25 3723–3729. 10.1093/ndt/gfq247 20466688

[B48] Jiang Y. Shen Z. Zhang J. Xing C. Zha X. Shen C. (2016). Parathyroidectomy increases heart rate variability and leptin levels in patients with stage 5 chronic kidney disease. *Am. J. Nephrol.* 44 245–254. 10.1159/000449018 27598394

[B49] Jourde-Chiche N. Dou L. Cerini C. Dignat-George F. Brunet P. (2011). Vascular incompetence in dialysis patients–protein-bound uremic toxins and endothelial dysfunction. *Semin. Dial.* 24 327–337. 10.1111/j.1525-139X.2011.00925.x 21682773

[B50] Kalra P. A. Green D. Poulikakos D. (2018). Arrhythmia in hemodialysis patients and its relation to sudden death. *Kidney Int.* 93 781–783. 10.1016/j.kint.2017.12.005 29571451

[B51] Kaur J. Young B. E. Fadel P. J. (2017). Sympathetic overactivity in chronic kidney disease: consequences and mechanisms. *Int. J. Mol. Sci.* 18:1682. 10.3390/ijms18081682 28767097 PMC5578072

[B52] Kentta T. Viik J. Karsikas M. Seppänen T. Nieminen T. Lehtimäki T. (2012). Postexercise recovery of the spatial QRS/T angle as a predictor of sudden cardiac death. *Heart Rhythm* 9 1083–1089. 10.1016/j.hrthm.2012.02.030 22387381

[B53] Lee A. S. Chen W. Y. Chan H. C. Chung C. H. Peng H. Y. Chang C. M. (2017). Electronegative LDL-mediated cardiac electrical remodeling in a rat model of chronic kidney disease. *Sci. Rep.* 7:40676. 10.1038/srep40676 28094801 PMC5240592

[B54] Levick S. P. Murray D. B. Janicki J. S. Brower G. L. (2010). Sympathetic nervous system modulation of inflammation and remodeling in the hypertensive heart. *Hypertension* 55 270–276. 10.1161/HYPERTENSIONAHA.109.142042 20048196 PMC2823485

[B55] Lin C. Y. Lin L. Y. Chen P. C. (2007). Analysis of T-wave morphology from the 12-lead electrocardiogram for prediction of long-term prognosis in patients initiating haemodialysis. *Nephrol. Dial. Transplant.* 22 2645–2652. 10.1093/ndt/gfm238 17452400

[B56] London G. M. Guérin A. P. Marchais S. J. Métivier F. Pannier B. Adda H. (2003). Arterial media calcification in end-stage renal disease: impact on all-cause and cardiovascular mortality. *Nephrol. Dial. Transplant.* 18 1731–1740. 10.1093/ndt/gfg414 12937218

[B57] Mainigi S. K. Chebrolu L. H. Romero-Corral A. Mehta V. Machado R. R. Konecny T. (2012). Prediction of significant conduction disease through noninvasive assessment of cardiac calcification. *Echocardiography* 29 1017–1021. 10.1111/j.1540-8175.2012.01752.x 22672350

[B58] Malik M. Buxton A. E. Huikuri H. Lombardi F. Schmidt G. Zabel M. (2018). Noninvasive electrophysiology in risk assessment and screening. *Heart Rhythm* 15 803–804. 10.1016/j.hrthm.2018.03.014 29857851

[B59] Matsuo H. Dohi K. Machida H. Takeuchi H. Aoki T. Nishimura H. (2018). Echocardiographic assessment of cardiac structural and functional abnormalities in patients with end-stage renal disease receiving chronic hemodialysis. *Circ. J.* 82 586–595. 10.1253/circj.CJ-17-0393 29093429

[B60] Ng H. Y. Hsueh S. K. Lee Y. T. Chiou T. T. Huang P. C. Lee C. T. (2017). Synergic impact of vascular calcification and low autonomic tone in mortality of hemodialysis patients. *Nephron* 137 91–98. 10.1159/000477827 28637038

[B61] Nowak K. L. Chonchol M. (2018). Does inflammation affect outcomes in dialysis patients? *Semin. Dial*. 31 388–397. 10.1111/sdi.12686 29513906 PMC6035073

[B62] Ogna A. Forni Ogna V. Mihalache A. Pruijm M. Halabi G. Phan O. (2015). Obstructive sleep apnea severity and overnight body fluid shift before and after hemodialysis. *Clin. J. Am. Soc. Nephrol.* 10 1002–1010. 10.2215/CJN.08760914 25862778 PMC4455216

[B63] Oikawa K. Ishihara R. Maeda T. Yamaguchi K. Koike A. Kawaguchi H. (2009). Prognostic value of heart rate variability in patients with renal failure on hemodialysis. *Int. J. Cardiol.* 131 370–377. 10.1016/j.ijcard.2007.10.033 18199499

[B64] Pedersen O. D. Abildstrom S. Z. Ottesen M. M. Rask-Madsen C. Bagger H. Køber L. (2006). Increased risk of sudden and non-sudden cardiovascular death in patients with atrial fibrillation/flutter following acute myocardial infarction. *Eur. Heart J.* 27 290–295. 10.1093/eurheartj/ehi629 16267070

[B65] Pei J. Tang W. Li L. X. Su C. Y. Wang T. (2015). Heart rate variability predicts mortality in peritoneal dialysis patients. *Ren. Fail.* 37 1132–1137. 10.3109/0886022X.2015.1061729 26123265

[B66] Perl J. Robinson B. Davies S. On behalf of the International Society for Peritoneal Dialysis Arbor Research Collaborative and the PDOPPS investigators (2014). Update on the peritoneal dialysis outcomes and practice patterns study (PDOPPS). *Perit. Dial. Int.* 34:332. 10.3747/pdi.2014.00162 24991047 PMC4079475

[B67] Poulikakos D. Banerjee D. Malik M. (2014a). Risk of sudden cardiac death in chronic kidney disease. *J. Cardiovasc. Electrophysiol.* 25 222–231. 10.1111/jce.12328 24256575

[B68] Poulikakos D. Malik M. Banerjee D. (2014b). Parathyroid hormone and heart rate variability in haemodialysis patients. *Nephron Clin. Pract.* 126 110–115. 10.1159/000360542 24686193

[B69] Poulikakos D. Malik M. Banerjee D. (2014c). Sex-dependent association between heart rate variability and pulse pressure in haemodialysis patients. *Nephron Clin. Pract.* 128 361–366. 10.1159/000368436 25502577

[B70] Poulikakos D. Ross L. Recio-Mayoral A. Cole D. Andoh J. Chitalia N. (2014d). Left ventricular hypertrophy and endothelial dysfunction in chronic kidney disease. *Eur. Heart J. Cardiovasc. Imaging* 15 56–61. 10.1093/ehjci/jet120 23811493

[B71] Poulikakos D. Banerjee D. Malik M. T. (2013). wave morphology changes during hemodialysis. *J. Electrocardiol.* 46 492–496. 10.1016/j.jelectrocard.2013.07.006 23972531

[B72] Poulikakos D. Hnatkova K. Banerjee D. Malik M. (2018). Association of QRS-T angle and heart rate variability with major cardiac events and mortality in hemodialysis patients. *Ann. Noninvasive Electrocardiol.* 23:e12570. 10.1111/anec.12570 29938866 PMC6931824

[B73] Poulikakos D. Malik M. (2016). Challenges of ECG monitoring and ECG interpretation in dialysis units. *J. Electrocardiol.* 49 855–859. 10.1016/j.jelectrocard.2016.07.019 27613393

[B74] Pun P. H. Hellkamp A. S. Sanders G. D. Middleton J. P. Hammill S. C. Al-Khalidi H. R. (2015). Primary prevention implantable cardioverter defibrillators in end-stage kidney disease patients on dialysis: a matched cohort study. *Nephrol. Dial. Transplant.* 30 829–835. 10.1093/ndt/gfu274 25404241 PMC4425479

[B75] Reeves P. B. Mc Causland F. R. (2018). Mechanisms, clinical implications, and treatment of intradialytic hypotension. *Clin. J. Am. Soc. Nephrol.* 13 1297–1303. 10.2215/CJN.12141017 29483138 PMC6086712

[B76] Ribas Ribeiro L. Flores de Oliveira J. Bueno Orcy R. Castilho Barros C. Damé Hense J. Santos F. (2018). Exploring the complexity: the interplay between the angiotensin-converting enzyme insertion/deletion polymorphism and the sympathetic response to hemodialysis. *Am. J. Physiol. Heart Circ. Physiol.* 315 H1002–H1011. 10.1152/ajpheart.00162.2018 29949384

[B77] Roberts P. R. Zachariah D. Morgan J. M. Yue A. M. Greenwood E. F. Phillips P. C. (2017). Monitoring of arrhythmia and sudden death in a hemodialysis population: the CRASH-ILR study. *PLoS One* 12:e0188713. 10.1371/journal.pone.0188713 29240772 PMC5730159

[B78] Robin J. Weinberg K. Tiongson J. Carnethon M. Reddy M. Ciaccio C. (2006). Renal dialysis as a risk factor for appropriate therapies and mortality in implantable cardioverter-defibrillator recipients. *Heart Rhythm* 3 1196–1201. 10.1016/j.hrthm.2006.06.013 17018351

[B79] Robinson E. A. Rhee K. S. Doytchinova A. Kumar M. Shelton R. Jiang Z. (2015). Estimating sympathetic tone by recording subcutaneous nerve activity in ambulatory dogs. *J. Cardiovasc. Electrophysiol.* 26 70–78. 10.1111/jce.12508 25091691 PMC4289443

[B80] Roy-Chaudhury P. Tumlin J. A. Koplan B. A. Costea A. I. Kher V. Williamson D. (2018). Primary outcomes of the monitoring in dialysis study indicate that clinically significant arrhythmias are common in hemodialysis patients and related to dialytic cycle. *Kidney Int.* 93 941–951. 10.1016/j.kint.2017.11.019 29395340

[B81] Sacher F. Jesel L. Borni-Duval C. De Precigout V. Lavainne F. Bourdenx J.-P. (2018). Cardiac rhythm disturbances in hemodialysis patients: early detection using an implantable loop recorder and correlation with biological and dialysis parameters. *JACC Clin. Electrophysiol.* 4 397–408. 10.1016/j.jacep.2017.08.002 30089568

[B82] Sakaguchi Y. Shoji T. Kawabata H. Niihata K. Suzuki A. Kaneko T. (2011). High prevalence of obstructive sleep apnea and its association with renal function among nondialysis chronic kidney disease patients in Japan: a cross-sectional study. *Clin. J. Am. Soc. Nephrol.* 6 995–1000. 10.2215/CJN.08670910 21415314 PMC3087795

[B83] Sallam K. Li Y. Sager P. T. Houser S. R. Wu J. C. (2015). Finding the rhythm of sudden cardiac death: new opportunities using induced pluripotent stem cell-derived cardiomyocytes. *Circ. Res.* 116 1989–2004. 10.1161/CIRCRESAHA.116.304494 26044252 PMC4676576

[B84] Sassi R. Cerutti S. Lombardi F. Huikuri H. V. Peng C. K. Schmidt G. (2015). Advances in heart rate variability signal analysis: joint position statement by the e-Cardiology ESC Working Group and the European Heart Rhythm Association co-endorsed by the Asia Pacific Heart Rhythm Society. *Europace* 17 1341–1353. 10.1093/europace/euv015 26177817

[B85] Semple D. Smith K. Bhandari S. Seymour A. M. (2011). Uremic cardiomyopathy and insulin resistance: a critical role for akt? *J. Am. Soc. Nephrol.* 22 207–215. 10.1681/ASN.2009090900 20634295

[B86] Severi S. Cavalcanti S. Avanzolini G. (1997). Heart rate variability spectral indices for haemodynamic classification of haemodialysis patients. *Physiol. Meas.* 18 339–353. 10.1088/0967-3334/18/4/007 9413867

[B87] Shang F. Wang S. C. Hsu C. Y. Miao Y. Martin M. Yin Y. (2017). MicroRNA-92a mediates endothelial dysfunction in CKD. *J. Am. Soc. Nephrol.* 28 3251–3261. 10.1681/ASN.2016111215 28696247 PMC5661278

[B88] Siddiqi L. Joles J. A. Grassi G. Blankestijn P. J. (2009). Is kidney ischemia the central mechanism in parallel activation of the renin and sympathetic system? *J. Hypertens.* 27 1341–1349. 10.1097/HJH.0b013e32832b521b 19444143

[B89] Silva R. T. Martinelli Filho M. Peixoto Gde L. Lima J. J. Siqueira S. F. Costa R. (2015). Predictors of arrhythmic events detected by implantable loop recorders in renal transplant candidates. *Arq. Bras. Cardiol.* 105 493–502. 10.5935/abc.20150106 26351983 PMC4651408

[B90] Simantirakis E. N. Schiza S. I. Marketou M. E. Chrysostomakis S. I. Chlouverakis G. I. Klapsinos N. C. (2004). Severe bradyarrhythmias in patients with sleep apnoea: the effect of continuous positive airway pressure treatment: a long-term evaluation using an insertable loop recorder. *Eur. Heart J.* 25 1070–1076. 10.1016/j.ehj.2004.04.017 15191779

[B91] Skampardoni S. Green D. Hnatkova K. Malik M. Kalra P. A. Poulikakos A. D. (2018a). QRS-T angle predicts Cardiac Risk and correlates with Global Longitudinal Strain in Prevalent Hemodialysis Patients. *Front. Physiol.* 10:145 10.3389/fphys.2019.00145 PMC6397862 30858805

[B92] Skampardoni S. Poulikakos D. Malik M. Green D. Kalra P. A. (2018b). The potential of electrocardiography for cardiac risk prediction in chronic and end-stage kidney disease. *Nephrol. Dial. Transplant.* 10.1093/ndt/gfy255 [Epub ahead of print]. 30085289 PMC6603366

[B93] Smetana P. Batchvarov V. N. Hnatkova K. Camm A. J. Malik M. (2002). Sex differences in repolarization homogeneity and its circadian pattern. *Am. J. Physiol. Heart Circ. Physiol.* 282 H1889–H1897. 10.1152/ajpheart.00962.2001 11959656

[B94] Suzuki M. Hiroshi T. Aoyama T. Tanaka M. Ishii H. Kisohara M. (2012). Nonlinear measures of heart rate variability and mortality risk in hemodialysis patients. *Clin. J. Am. Soc. Nephrol.* 7 1454–1460. 10.2215/CJN.09430911 22723446 PMC3430947

[B95] Tada T. Kusano K. F. Ogawa A. Iwasaki J. Sakuragi S. Kusano I. (2007). The predictors of central and obstructive sleep apnoea in haemodialysis patients. *Nephrol. Dial. Transplant.* 22 1190–1197. 10.1093/ndt/gfl748 17277346

[B96] Tang W. Li L. X. Pei J. Wang T. (2012). Heart rate variability in peritoneal dialysis patients: what is the role of residual renal function? *Blood Purif.* 34 58–66. 10.1159/000338184 22922790

[B97] Task Force of the European Society of Cardiology and North American Society of Pacing and Electrophysiology (1996). Heart rate variability: standards of measurement, physiological interpretation and clinical use. *Circulation* 93 1043–1065. 8598068

[B98] Tereshchenko L. G. Kim E. D. Oehler A. Meoni L. A. Ghafoori E. Rami T. (2016). Electrophysiologic substrate and risk of mortality in incident hemodialysis. *J. Am. Soc. Nephrol.* 27 3413–3420. 10.1681/ASN.2015080916 27129390 PMC5084888

[B99] Tsuji Y. Suzuki N. Hitomi Y. Yoshida T. Mizuno-Matsumoto Y. (2017). Quantification of autonomic nervous activity by heart rate variability and approximate entropy in high ultrafiltration rate during hemodialysis. *Clin. Exp. Nephrol.* 21 524–530. 10.1007/s10157-016-1305-5 27480095

[B100] United States Renal Data System (2012). *USRDS Annual Data Report: Epidemiology of Kidney Disease in the United States.* Bethesda, MD: National Institutes of Health.

[B101] Vallon V. Muhlbauer B. Osswald H. (2006). Adenosine and kidney function. *Physiol. Rev.* 86 901–940. 10.1152/physrev.00031.2005 16816141

[B102] Vaseghi M. Lux R. L. Mahajan A. Shivkumar K. (2012). Sympathetic stimulation increases dispersion of repolarization in humans with myocardial infarction. *Am. J. Physiol. Heart Circ. Physiol.* 302 H1838–H1846. 10.1152/ajpheart.01106.2011 22345568 PMC3362058

[B103] Vitolo E. Madoi S. Palvarini M. De Maria R. Ciró E. Colombo A. E. (1987). Relationship between changes in R wave voltage and cardiac volumes. A vectorcardiographic study during hemodialysis. *J. Electrocardiol.* 20 138–146. 10.1016/S0022-0736(87)80103-6 3598455

[B104] Waks J. W. Sitlani C. M. Soliman E. Z. Kabir M. Ghafoori E. Biggs M. L. (2016). Global electric heterogeneity risk score for prediction of sudden cardiac death in the general population: the atherosclerosis risk in communities (ARIC) and cardiovascular health (CHS) studies. *Circulation* 133 2222–2234. 10.1161/CIRCULATIONAHA.116.021306 27081116 PMC4899170

[B105] Wan C. Herzog C. A. Zareba W. Szymkiewicz S. J. (2014). Sudden cardiac arrest in hemodialysis patients with wearable cardioverter defibrillator. *Ann. Noninvasive Electrocardiol.* 19 247–257. 10.1111/anec.12119 24252154 PMC4034590

[B106] Wang A. Y. Wang M. Woo J. Lam C. W. Li P. K. Lui S. F. (2003). Cardiac valve calcification as an important predictor for all-cause mortality and cardiovascular mortality in long-term peritoneal dialysis patients: a prospective study. *J. Am. Soc. Nephrol.* 14 159–168. 10.1097/01.ASN.0000038685.95946.83 12506148

[B107] Wang I. K. Lin K. H. Lin S. Y. Lin C. L. Chang C. T. Yen T. H. (2016). Permanent cardiac pacing in patients with end-stage renal disease undergoing dialysis. *Nephrol. Dial. Transplant.* 31 2115–2122. 10.1093/ndt/gfw302 27540047

[B108] Wang Z. Jiang A. Wei F. Chen H. (2018). Cardiac valve calcification and risk of cardiovascular or all-cause mortality in dialysis patients: a meta-analysis. *BMC Cardiovasc. Disord.* 18:12. 10.1186/s12872-018-0747-y 29370754 PMC5785897

[B109] Wanner C. Krane V. März W. Olschewski M. Mann J. F. Ruf G. (2005). Atorvastatin in patients with type 2 diabetes mellitus undergoing hemodialysis. *N. Engl. J. Med.* 353 238–248. 10.1056/NEJMoa043545 16034009

[B110] Wolf J. Drozdowski J. Czechowicz K. Winklewski P. J. Jassem E. Kara T. (2016). Effect of beta-blocker therapy on heart rate response in patients with hypertension and newly diagnosed untreated obstructive sleep apnea syndrome. *Int. J. Cardiol.* 202 67–72. 10.1016/j.ijcard.2015.08.139 26386925 PMC4656106

[B111] Wong M. C. Kalman J. M. Pedagogos E. Toussaint N. Vohra J. K. Sparks P. B. (2015). Temporal distribution of arrhythmic events in chronic kidney disease: highest incidence in the long interdialytic period. *Heart Rhythm* 12 2047–2055. 10.1016/j.hrthm.2015.06.033 26111801

[B112] Wong M. C. G. Kalman J. M. Pedagogos E. Toussaint N. Vohra J. K. Sparks P. B. (2015). Bradycardia and asystole is the predominant mechanism of sudden cardiac death in patients with chronic kidney disease. *J. Am. Coll. Cardiol.* 65 1263–1265. 10.1016/j.jacc.2014.12.049 25814235

[B113] Young C. N. Fisher J. P. Gallagher K. M. Whaley-Connell A. Chaudhary K. Victor R. G. (2009). Inhibition of nitric oxide synthase evokes central sympatho-excitation in healthy humans. *J. Physiol.* 587(Pt 20) 4977–4986. 10.1113/jphysiol.2009.177204 19723781 PMC2770160

[B114] Zabel M. Malik M. (2002). Practical use of T wave morphology assessment. *Card. Electrophysiol. Rev.* 6 316–322. 10.1023/A:1016353714372 12114858

[B115] Zhang J. Yu X. Sun B. Bai J. Wei Y. Zha X. (2013). Parathyroidectomy and heart rate variability in patients with stage 5 CKD. *Clin. J. Am. Soc. Nephrol.* 8 1378–1387. 10.2215/CJN.10381012 23660181 PMC3731907

[B116] Zhang L. N. Yang G. Cheng C. Shen C. Cui Y. Y. Zhang J. (2015). Plasma FGF23 levels and heart rate variability in patients with stage 5 CKD. *Osteoporos. Int.* 26 395–405. 10.1007/s00198-014-2862-7 25224292

[B117] Zhao Y. Chen N. X. Shirazi J. T. Shen C. Lin S. F. Fishbein M. C. (2016). Subcutaneous nerve activity and mechanisms of sudden death in a rat model of chronic kidney disease. *Heart Rhythm* 13 1105–1112. 10.1016/j.hrthm.2015.12.040 26744093 PMC4851901

[B118] Zwillich C. Devlin T. White D. Douglas N. Weil J. Martin R. (1982). Bradycardia during sleep apnea. Characteristics and mechanism. *J. Clin. Invest.* 69 1286–1292. 10.1172/JCI110568 7085875 PMC370201

